# Case report: A novel missense variant in melanopsin associates with delayed sleep phenotype: Whole genome sequencing study

**DOI:** 10.3389/fgene.2022.896192

**Published:** 2022-09-30

**Authors:** Sandra P. Smieszek, Christos M. Polymeropoulos, Gunther Birznieks, Mihael H. Polymeropoulos

**Affiliations:** Vanda Pharmaceuticals Inc, Washington, DC, United States

**Keywords:** melanopsin (OPN4), DSWPD, circadian clock, risk locus, sequencing

## Abstract

Melanopsin (OPN4) is a blue light-sensitive opsin-type G-protein coupled receptor. It is highly expressed in photosensitive retinal ganglion cells which mediate responses to light, including regulation of sleep, circadian photoentrainment, and pupillary light response. Mutations in *OPN4* were shown to affect responses to light, ultimately affecting the regulation of circadian rhythms and sleep. In this study, we describe a male carrier of the *OPN4* missense variant diagnosed with delayed sleep-wake phase disorder (DSWPD), with a consistent recurrent pattern of delayed sleep onset The rs143641898 [NM_033282.4:c.502C>T p.(Arg168Cys)] variant in the *OPN4* gene was shown in a functional study to render the OPN4 protein non-functional. The variant is rare and likely increases the risk of DSWPD *via* its direct effect on the melanopsin pathway. This study offers useful insights for the differential diagnosis and ultimately treatment of DSWPD risk in which patients carry pathogenic variants in the *OPN4* gene.

## Brief communication

Melanopsin (OPN4) is a blue light-sensitive opsin-type G-protein coupled receptor (Provencio et al., 2000). It is highly expressed in photosensitive retinal ganglion cells that mediate responses to light including regulation of sleep, circadian photoentrainment, and pupillary light response (Panda et al., 2002; Rodgers et al., 2018). Consequential variants in OPN4 were previously associated with an increased risk of developing the seasonal affective disorder (Chellappa, 2021). Melanopsin-dependent phototransduction was reported to be impaired in DSWPD and sighted non-24-hour sleep-wake rhythm disorder (Abbott et al., 2021). DSWPD is the most commonly diagnosed circadian rhythm sleep-wake disorder, with an estimated prevalence of 0.2%–10% (Nesbitt, 2018; Zee et al., 2013). It is characterized by a persistent and intractable delay in sleep onset and offset times relative to the societal norm (Zee et al., 2013). In this case report, we describe a case study of a DSWPD patient with a consequential damaging variant in *OPN4*.

We report a carrier of a rare variant: rs143641898 [NM_033282.4:c.502C>T p.(Arg168Cys)], a rare missense variant (gnomAD (Karczewski et al., 2019) MAF 0.0002). It is most common among European (non-Finnish) and Ashkenazi Jewish populations and extremely rare among East Asian (0.00005) and not detected in South Asian populations, suggesting the highest conservation of this region of the gene across these populations. It is *in silico* predicted to be damaging and has a Combined Annotation Dependent Depletion (CADD) score of 34. It is a highly conserved variant, and it is a part of the E/DRY motif found in nearly all GPCRs (positive charge to polar, increased hydrophobicity). Interestingly, this variant (rs143641898) was tested using an *in vitro* expression system as part of a study aiming to determine the functional phenotypes of missense human *OPN4* variants (Rodgers et al., 2018). The authors selected 16 potentially deleterious variants for functional characterization using calcium imaging of melanopsin-driven light responses in HEK293T cells (Rodgers et al., 2018). This variant was shown to be incapable of binding retinal chromophore, suggesting that it renders the OPN4 protein non-functional (Rodgers et al., 2018). The introduction of rs143641898 in *OPN4* abolished responses to light.

We do not detect this variant in our healthy sleeping super control set of whole genome sequencing (WGS) samples (*n* = 300) as well as in our control set of WGS (*n* = 1900). In our whole genome sequencing study of DSWPD patients, we report one other rare stop-gain in *OPN4* [NM_033282.4:c.1086_1087insTAGCGG p.(Gln363*)] variant within the *OPN4* gene. This variant has been detected in an unrelated individual. This variant requires functional confirmation similar to the one that was carried out for rs143641898.

**FIGURE 1:**
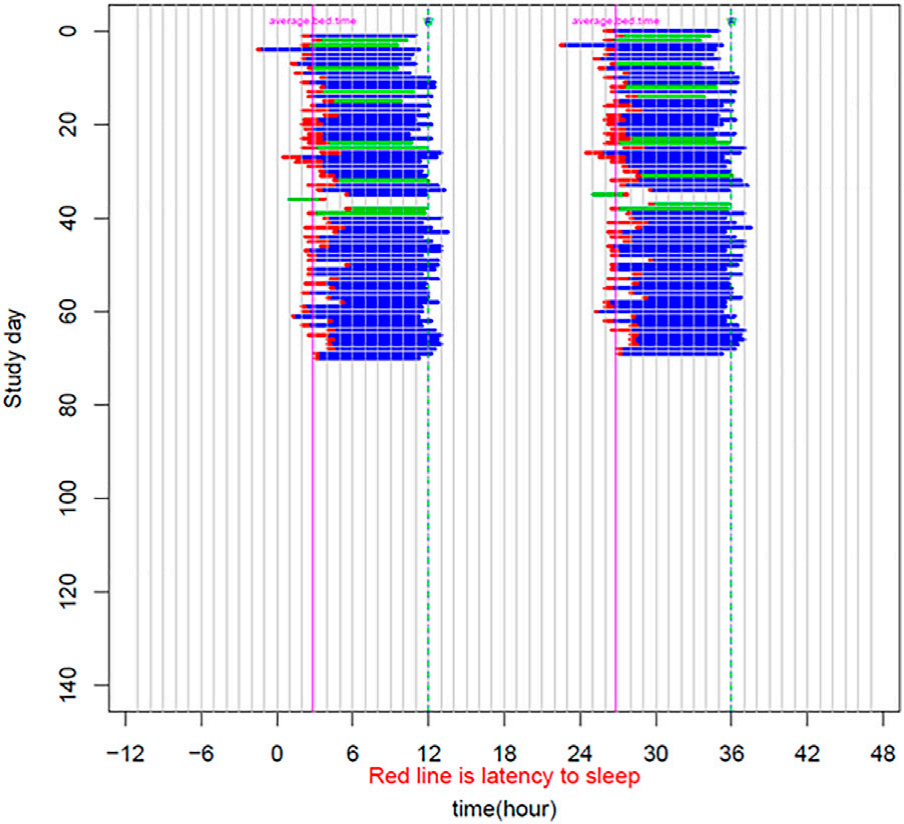
Sleep raster plot showing sleep timing (sleep onset, duration, and wake-up time) as measured with an electronic sleep diary over a period of 70 days in the carrier of the rs143641898 *OPN4* variant. The raster plot confirms consistently delayed sleep onset captured over more than 10 weeks of a sleep diary.

**FIGURE 2:**
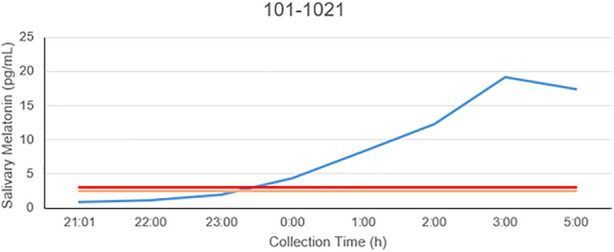
Salivary melatonin DLMO in the carrier of the rs143641898 *OPN4* variant. The DLMO is significantly later than that of population controls, in this example, occurring at 23:17.

The patient is a 58-year-old male manifesting DSWPD symptoms since high school. The patient meets DSWPD diagnostic criteria (DSWPD—ICSD-3 (Sateia, 2014) based on a clinical interview with a board-certified sleep physician). The patients’ medical records have an earlier diagnosis of insomnia (per medical history). The patient has been enrolled in a 12-week diary study (Figure 1 raster plot below). The reported sleep onset is consistent and significantly delayed in comparison to a control population. The patient reported an average bedtime of 2:48 a.m., average sleep onset of 3:29 a.m., and average wake-up time of 11:42 a.m. (average over 12 weeks of electronic time-stamped sleep diary). The patient reported a consistent total sleep time (TST) of 8:14 (average over 12 weeks). Confirmatory of the delayed phenotype is also the timing of the dim light melatonin onset (DLMO) occurring at 23:20 (quantified with the salivary melatonin assay) presented in Figure 2. The patient has no other known pLOF mutations reported in the core circadian clock genes including no variants or copy number variants detected in the *CRY1* gene or in the *AANAT* gene. Variants within both were previously associated with DSWPD, and despite the fact they are highly penetrant, they do not explain a large proportion of individuals with DSWPD of mixed ancestry (Patke et al., 2017; Hohjoh et al., 2003; Smieszek et al., 2021).

These data provide valuable insights into the phenotype-genotype consequences of human *OPN4* variants. Additional studies focusing on a set of carriers of this and other damaging variants in *OPN4* are warranted to confirm this finding. One example would be looking at the lab-based assessment of plasma melatonin suppression under 460 nm (blue) and 555 nm monochromatic light would be very interesting for this patient, as well as other carriers of this variant. Given melanopsin is not functional, there would be little or no suppression in the last half to quarter of the 460 nm light, which is all melanopsin driven; however, the 555 nm (cone system) would look normal (and the opposite to the blind man who only has melanopsin). Additionally, studies looking at pupil constriction dynamics could be helpful confirmatory studies (Gooley et al., 2012). The identification of consequential *OPN4* variants leading to disruption of protein function such as rs143641898 leading to disrupted melanopsin-based light perception will help with the identification of patients with an increased risk of sleep disturbances and circadian dysfunction, who may need early interventions.

## Methods

### Electronic sleep diary

Participants reported their bedtime and wake time in a daily sleep diary. Daily diaries were collected for up to 12 weeks (a minimum of 4 weeks). Data were summarized with means, medians, SDs, minimums, and maximums. The mean was calculated as the average of individual means. The individual mean was calculated as the average for each participant over all nights, work nights, and free nights. For SD, the mean of individual SD was calculated. Analyses were carried out for both work nights and free nights, defined as a night before a work/morning commitment and a night before a free day, respectively.

### Whole genome sequencing

Incoming nucleic acid samples are quantified using fluorescent-based assays (PicoGreen) to accurately determine whether sufficient material is available for library preparation and sequencing. DNA sample size distributions are profiled by a Fragment Analyzer (Advanced Analytics) or BioAnalyzer (Agilent Technologies) to assess sample quality and integrity. Whole genome sequencing (WGS) libraries were prepared using the Truseq DNA PCR-free Library Preparation Kit. Whole genome data were processed on an automated pipeline by the New York Genome Center. Paired-end 150 bp reads were aligned to the GRCh37 human reference [BWA-MEM (Li and Durbin, 2009) v0.7.8] and processed with the GATK best-practices workflow [GATK v3.4.0 (van der Auwera et al., 2013)]. The mean coverage was 35.8. All high-quality variants obtained from GATK were annotated for functional effects (*intronic*, *intergenic*, *splicing*, *nonsynonymous*, *stop-gain*, *and frameshifts*) based on RefSeq transcripts using Annovar (Wang et al., 2010). Additionally, Annovar was used to match general population frequencies from public databases (EXAC, gnomAD, ESP6500, 1000 g) and to prioritize pLOFs. The analysis focused on rare and common consequential OPN4 variants such as missense, frameshift, and splicing variants.

### Salivary dim light melatonin onset assessment kit

Salimetrics’ saliva collection devices, assay kits, and CLIA-certified testing services were used. This is an ELISA-based assay with a sensitivity of 1.37 pg/ml and an assay range of 0.78–50 pg/ml. The DLMO assessment consisted of eight scheduled saliva collections to be performed beginning from 5 h before bedtime until 3 h after bedtime.

## Data Availability

The raw data supporting the conclusions of this article will be made available by the authors, without undue reservation.
